# Diagnostic Challenges and Management of Acquired Hemophilia A Presenting as Gross Hematuria: A Case Report

**DOI:** 10.7759/cureus.51426

**Published:** 2023-12-31

**Authors:** Aris Kaltsas, Athanasios Zachariou, Zisis Kratiras, Nikolaos Sofikitis, Michael Chrisofos

**Affiliations:** 1 Third Department of Urology, Attikon University Hospital, School of Medicine, National and Kapodistrian University of Athens, Athens, GRC; 2 Department of Urology, University of Ioannina, Ioannina, GRC

**Keywords:** autoimmune disorder, coagulation disorders, factor viii inhibitor, gross hematuria, acquired hemophilia a

## Abstract

Acquired hemophilia A (AHA) is a rare autoimmune disorder marked by autoantibodies against coagulation factor VIII, leading to bleeding complications. This case report explores a unique presentation of AHA, initially manifested as gross hematuria, a symptom often encountered in healthcare settings with a broad range of differential diagnoses. The background of this study highlights the rarity of AHA and its diverse clinical presentations. The case involves a 62-year-old man with no history of bleeding disorders, presenting with gross hematuria and later developing severe anemia and ecchymoses. Methods employed in the evaluation included urological assessments such as cystoscopy and computed tomography, alongside hematological investigations, which later revealed a prolonged activated partial thromboplastin time (aPTT) and a critically low factor VIII level, indicative of AHA. Results showed a lack of early recognition of coagulation abnormalities, underscoring the need for comprehensive initial assessments in cases of unexplained hematuria. The patient's management at a specialized Hemophilia Center involved inhibitor eradication therapy and management of acute bleeding episodes, resulting in significant clinical improvement. The conclusions drawn from this case emphasize the importance of considering rare conditions like AHA in the differential diagnosis of hematuria and the necessity for a broad diagnostic approach. It advocates for heightened awareness and early coagulation studies in unexplained cases of hematuria to prevent delayed diagnoses and improve patient outcomes. This case contributes to the understanding of AHA's clinical variability and the critical nature of early and comprehensive diagnostic approaches in hematuria evaluation.

## Introduction

Acquired hemophilia A (AHA), also known as acquired factor VIII inhibitor, is a rare autoimmune disorder characterized by the presence of autoantibodies against coagulation factor VIII (FVIII). These autoantibodies disrupt the normal functioning of FVIII within the coagulation cascade, leading to bleeding problems. Factor VIII functions as a cofactor for factor IXa, facilitating the activation of factor X (FX) in the intrinsic pathway. The failure to activate FX, and consequently hindered thrombin synthesis, manifests in various clinical forms of bleeding [1-3].

Reflecting its rarity, with an annual incidence of just 1 per 1,000,000 in the general population, this condition presents with a range of bleeding manifestations, including ecchymosis, hematomas, gastrointestinal bleeding, hematuria, and intracerebral hemorrhages [3]. Hematuria, representing mucosal bleeding in the genitourinary system, is a symptom observed in various pathological conditions, leading to a broad range of differential diagnoses [4]. This diversity often results in diagnostic challenges and potential delays in identifying rare diseases that manifest with common symptoms. In half of the cases, AHA is secondary to conditions such as malignancy, autoimmune diseases, or pregnancy. However, in the other 50% of patients, no accompanying condition is identified, and the disorder is deemed idiopathic [5]. The complexity of diagnosing AHA is highlighted in cases where common symptoms mask the underlying rare condition, emphasizing the importance of thorough evaluation in patients presenting with unexplained bleeding symptoms.

## Case presentation

A 62-year-old man with no significant personal or family history of bleeding disorders was admitted for medical evaluation, presenting with gross hematuria, where blood was visibly present in the urine along with multiple clots. The presence of clots, which posed a risk for urinary retention and further complications, necessitated immediate intervention. To manage this, bladder irrigation was initiated, utilizing a continuous irrigation method through a 24 Fr. three-way catheter.

The laboratory results revealed a hemoglobin level of 9.5 g/dL, a hematocrit value of 28.8%, a prothrombin time (PT) of 13.1 seconds, a normal platelet count of 400 x 10^9/L, and an international normalized ratio (INR) of 0.99 and a prolonged activated partial thromboplastin time (aPTT) of 69.5 seconds. The issue of prolonged aPTT was not addressed throughout that period.

We proceeded to investigate hematuria by cystoscopy and computed tomography (CT). During the cystoscopy, we observed the ureteral orifices closely. While there was no evidence of intrinsic bladder pathology, we noted features consistent with benign prostatic hyperplasia (BPH), including the presence of a median lobe. This finding was significant as BPH, particularly with a prominent median lobe, can be a contributory factor for hematuria because of increased vascularization and potential for bleeding. 

Subsequently, a CT scan was performed to assess the kidneys and ureters for any abnormalities that might explain the hematuria. The CT results indicated the absence of any abnormalities or pathological conditions in the kidneys or ureters. Combined with the cystoscopic findings, this led us to conclude that the likely source of the hematuria was related to the patient's prostate condition, specifically the benign hyperplasia with the presence of a median lobe.

Considering this, while the appearance of the urine at the ureteral orifices did not indicate a renal origin of the hematuria, the presence of BPH with a median lobe provided a plausible explanation for the patient's condition. This diagnosis is consistent with the known association between BPH and hematuria, where enlarged prostate tissue and increased vascularity can lead to bleeding episodes.

On the seventh day of hospitalization, there was a notable progression in the patient's condition. He exhibited extensive ecchymoses and hematomas, predominantly affecting his extremities. Concurrently, his laboratory results showed normal PT/INR values, but a concerningly prolonged aPTT of 128.6 seconds was observed. These clinical signs indicated a severe bleeding disorder, prompting a thorough hematological evaluation by the hematology department.

As part of the coagulation investigation, a detailed analysis was conducted. This included a mixing study, lupus anticoagulant assessment, and measurement of inhibitor levels for factors VIII, IX, and XI. The lupus anticoagulant test results were negative, and the patient was found to have deficient iron levels, necessitating the initiation of intravenous iron therapy. This unique combination of findings, particularly the unadjusted clotting time, pointed towards the likely presence of an inhibitor. Further testing for heparin-induced thrombocytopenia (HIT) antibodies came back negative. Confirmatory tests for factor VIII inhibitors showed a critically low FVIII level of less than 1%, a finding indicative of either a severe deficiency or the presence of an inhibitor. The patient was then transferred to the Hemophilia Center of Greece for specialized care.

At the Hemophilia Center, further diagnostic tests confirmed the presence of an FVIII inhibitor, establishing a diagnosis of AHA. Unfortunately, during the evaluation, the patient experienced clinical deterioration, presenting with intra-abdominal bleeding and compartment syndrome in his left forearm. His hemoglobin level dropped significantly to 5 g/dL.

The treatment regimen included inhibitor eradication therapy with corticosteroids, cyclophosphamide, and rituximab, alongside management of acute bleeding episodes with recombinant factor VIIa (rFVIIa) and transfusions of packed red blood cells and plasma transfusions to provide additional supportive care. Over the following weeks, the patient showed gradual improvement with stabilization of hemoglobin levels and subsidence of bleeding episodes. The hematuria ceased following the successful implementation of inhibitor eradication therapy. Once the bleeding was controlled and the hematuria had stopped, the Foley catheter was removed. Follow-up tests revealed a significant recovery in FVIII levels, increasing to 65%.

Upon discharge, the patient was provided with a comprehensive plan to taper off corticosteroids and was scheduled for regular follow-up appointments. To provide a clearer overview of the patient's coagulation profile and bleeding control at different stages of his hospital stay, Table 1 summarizes key laboratory findings on the day of admission, on the third and seventh days of hospitalization, and at a three-month follow-up. This case underscores the importance of considering rare conditions such as AHA in patients presenting with nonspecific symptoms such as hematuria and highlights the challenges in diagnosing and managing such complex cases.

**Table 1:** Analysis of Coagulation and Bleeding Control at Different Time Points

Laboratory analysis	Units	Reference Range	Day 1 (Admission)	Day 3	Day 7	3 Month Follow-up
Mean PT	seconds	11-13.5	13.2	13.2	12.8	12.7
Patient PT	seconds	11-13.5	13.1	13.3	12.5	11.9
I.N.R.	ratio	0.8-1.2	0.99	1.01	1.02	0.94
Mean aPTT	seconds	24-35	28.0	28.0	28.0	28.0
Patient aPTT	seconds	24-35	69.5	72.3	128.6	29.9

## Discussion

The evaluation of hematuria, a fundamental component of urological practice, requires urologists to adopt a comprehensive approach to diagnosing and managing this clinical presentation. Hematuria, whether it presents in a microscopic or gross form, demands an in-depth assessment to elucidate its underlying etiology, which spans a spectrum from benign conditions to potentially serious urological malignancies [6]. This process involves meticulous collection of clinical history, detailed physical examinations, and the deployment of diverse diagnostic investigations. The primary objective is to ascertain the root cause of hematuria and facilitate informed decisions regarding patient management [7].

The role of urologists in evaluating hematuria is underscored by its significance as a potential early marker of urological malignancies, including bladder cancer and renal cell carcinoma. Urologists employ a variety of diagnostic modalities, such as cystoscopy, imaging studies, and urine cytology, to accurately identify the source and characteristics of hematuria, thereby informing appropriate management strategies. Additionally, the advent of novel technologies, particularly urine-based molecular markers, presents a valuable opportunity to refine diagnostic precision in hematuria evaluations, which may lead to improved patient outcomes [6,7].

A critical aspect of hematuria evaluation is the recognition and addressing of gender disparities in referral patterns and diagnostic methods. Studies have illuminated significant variances in the approach toward the evaluation and management of hematuria among male and female patients. Efforts are currently underway to standardize evaluation processes and develop comprehensive risk stratification models, with the goal of providing equitable, unbiased, and evidence-based care to all patients, irrespective of gender [8,9].

The AHA as a rare disease manifesting as persistent gross hematuria encompasses the clinical challenges and management strategies associated with this unique presentation. AHA, characterized by the development of autoantibodies against coagulation factor VIII, typically presents with spontaneous hemorrhage, making its manifestation as persistent gross hematuria an unusual and diagnostically challenging scenario [1]. The optimal management of AHA requires a multidisciplinary approach, involving close collaboration between physicians from various specialties to ensure accurate diagnosis and appropriate treatment [10].

The case report of AHA presenting as persistent gross hematuria underscores the importance of considering AHA in the differential diagnosis of unexplained bleeding, especially in atypical presentations [11]. This highlights the need for increased awareness among healthcare professionals on the diverse clinical manifestations of AHA, as early recognition is crucial for timely intervention and improved patient outcomes [1]. Furthermore, it deserves to be highlighted that AHA should be suspected in cases of sudden, persistent, unexplained hematuria or other bleeding events in elderly patients without anticoagulant use, known personal or family history of bleeding, with comorbidities, or postpartum patients, especially if combined with aPTT prolongation [12]. Additionally, it deserves to be mentioned that even after successful treatment of the patient, long-term monitoring is necessary because of the observed relapse in 12%-18% of patients [13].

Furthermore, the successful use of novel therapeutic options, such as emicizumab, in refractory AHA cases with comorbidities, emphasizes the evolving treatment landscape for this rare bleeding disorder [14]. The emergence of targeted therapies and potential role of immunomodulatory agents, including rituximab, in achieving remission in AHA cases associated with autoimmune conditions, such as systemic lupus erythematosus, further expands the armamentarium for managing this challenging disease [15].

The discussion also delves into the association of AHA with other medical conditions, such as hepatitis C virus infection and rheumatoid arthritis, highlighting the complex interplay between autoimmune phenomena and the pathogenesis of AHA [16,17]. Additionally, the rarity of acquired hemophilia B through liver transplantation underscores the need for vigilance in monitoring and managing coagulation disorders in unique clinical contexts [18].

## Conclusions

Hematuria is a common presenting sign in various healthcare settings, and its initial evaluation typically includes assessing potential infectious causes, conducting imaging studies, and consulting with a urologist to eliminate urological disorders. However, it is crucial to consider less common medical illnesses in the differential diagnosis. For instance, in the case of a patient who initially presented with gross hematuria and underwent urologic assessment, the significant finding of a prolonged aPTT was overlooked. This oversight became critical when the patient later developed severe anemia, numerous ecchymoses, and persistent hematuria, necessitating critical care intervention.

Current guidelines from the American Urological Association for the first assessment of hematuria do not routinely include coagulation investigations. This raises the need for further research to evaluate the potential benefits of early coagulation studies in patients presenting with hematuria. Such research could provide valuable insights into whether early identification of coagulation abnormalities could improve patient outcomes. Moreover, in cases where there is an isolated and unexplained prolongation of aPTT combined with macroscopic hematuria, it is imperative to consider AHA in the differential diagnosis. Early detection of this serious and potentially fatal condition is critical for the timely initiation of appropriate treatment, which can significantly improve patient survival. This underscores the importance of broadening the diagnostic approach and engaging in innovative thinking to prevent delayed diagnosis and enhance patient care outcomes. Given the implications of this single case study, further investigation is necessary to fully understand the clinical significance and to inform future practice.
